# Effects of vitamin K supplementation on vascular calcification in chronic kidney disease: A systematic review and meta-analysis of randomized controlled trials

**DOI:** 10.3389/fnut.2022.1001826

**Published:** 2023-01-10

**Authors:** Chanyu Geng, Liming Huang, Lei Pu, Yunlin Feng

**Affiliations:** ^1^Department of Nephrology, Sichuan Academy of Medical Sciences and Sichuan Provincial People’s Hospital, Chengdu, China; ^2^Internal Medicine, School of Medicine, University of Electronic Science and Technology of China, Chengdu, China

**Keywords:** vitamin K, vascular calcification, chronic kidney disease, systematic review, meta-analysis

## Abstract

**Background:**

There is conflicting data on the effect of vitamin K supplementation against vascular calcification in chronic kidney disease (CKD). We aimed to summarize current evidence from randomized controlled trials (RCTs) to determine whether vitamin K supplementation in CKD could attenuate vascular calcification.

**Methods:**

A systematic search was performed in MEDLINE, EMBASE, and Cochrane Central Library. RCTs assessing the effect of vitamin K supplementation on vascular calcification in CKD and reported measures relevant to vascular calcification were eligible for inclusion. Effect outcomes are changes of biochemical and imaging measures of vascular calcification, as well as vascular elasticity reflected by pulse wave velocity (PWV). Safety outcomes included any adverse event and death. The risk of bias was assessed according to Cochrane handbook guidelines. Mean differences or standardized mean differences (SMD) with 95% confidence intervals (CIs) of absolute and relative changes of each studied outcome between experimental and control groups were pooled using a random-effects model.

**Results:**

In all, ten RCTs with 733 patients were included. Pooled results indicated a decrease in serum biomarkers relevant to vascular calcification to a certain extent, mild improvement in vascular elasticity reflected by PWV, yet, no significant change in calcification scores derived from radiology examinations. Half of the included studies had low risk of bias.

**Conclusion:**

Therefore, there is not yet solid evidence to support protective effects of vitamin K supplementation against vascular calcification in CKD. The results of ongoing RCTs are needed to further elucidate the value of vitamin K in this field.

**Systematic review registration:**

www.crd.york.ac.uk/prospero, identifier CRD42022343857.

## Introduction

Vascular calcification is an important and challenging complication of chronic kidney disease (CKD) and has been proven as an independent risk factor for patients’ mortality and poor quality of life (1). Strategies that may attenuate or delay the progression of vascular calcification in CKD have gained great attention yet remain uncertain (2).

The theoretical effect of vitamin K to attenuate vascular calcification began with the observation that patients who had been treated by warfarin had more serious burden of vascular calcification than those who had not (3). Later studies revealed vitamin K deficiency induced dysfunction of gamma-carboxyglutamic acid (Gla) proteins that widely distribute in vessels and skeleton might cause impaired vascular metabolism, leading to accelerated vascular calcification (4). The prevalence of vitamin K deficiency in the hemodialysis (HD) population (5, 6) and close relationship between vitamin K deficiency and vascular calcification in HD patients (7) were further confirmed. Many studies have explored the effect of vitamin K supplementation to protect patients against vascular calcification, however, there is conflicting or insufficient data in CKD population (2).

Therefore, we conducted this systematic review and meta-analysis to summarize current evidence on the effects of vitamin K supplementation on vascular calcification in CKD, to determine whether vitamin K supplementation could attenuate vascular calcification and help further research and clinical practice on this topic.

## Materials and methods

### Data sources and searches

A systematic search according to the Preferred Reporting Items for Systematic Review and Meta-Analyses statement (8) was performed for eligible studies published up to 1st July, 2022 in the following data sources: MEDLINE *via* PubMed (from 1946 through July 2022), EMBASE (from 1980 through July 2022), and Cochrane Central Library (no date restriction). The search strategy used text words and medical subject headings relevant to vitamin K and CKDs (see Supplementary Table 1). The search was limited to publication in English. The study was registered on PROSPERO (Identifier# CRD42022343857).

### Study selection

Randomized controlled trials (RCTs) that had assessed the effects of vitamin K supplementation on vascular calcification in CKDs and reported measures relevant to vascular calcification were considered eligible for inclusion. Vitamin K could be either vitamin K1 or K2.

Two reviewers (G.C.Y. and H.L.M.) independently conducted the review process following a standardized approach. Titles and abstracts of all returned records were carefully reviewed. Duplications, non-original studies (e.g., guidelines, reviews, opinions, and editorial commentaries), non-human studies, vitamin K studies in non-CKD populations, and vitamin K studies that had not reported vascular calcification indexes were excluded. Reference lists from full text reviewed articles were further manually scanned to identify any other relevant studies. Any discrepancy was adjudicated by a third reviewer (F.Y.L.).

### Outcomes

Effect outcomes considered in this meta-analysis can be divided into three categories. The first category was absolute and relative changes of known biochemical measures relevant to vascular calcification, including osteocalcin (OC) and dephosphorylated uncarboxylated matrix Gla protein (dp-ucMGP). The second category was absolute and relative changes of imaging measures of vascular calcification, including Agaston scores of coronary artery, aortic artery, and valve as well as calcification volume scores of coronary artery and valve. The third category was vascular elasticity reflected by pulse wave velocity (PWV). In addition, information on safety outcomes was also collected, including any adverse event and death.

### Data extraction and quality assessment

Two reviewers (G.C.Y. and H.L.M.) independently extracted data from eligible studies following a double-check procedure and compiled them into a shared sheet. Disagreements were resolved by the third reviewer (F.Y.L.). The data extracted included authors, year of publication, identifier number if registered, geographical origin, number of participants, details of vitamin K supplementation, details of control, studied outcomes, and adverse events. Information about potential sources of significant clinical heterogeneity, such as composition of participants, was also collected for potential sensitivity analysis.

### Critical appraisal of included studies

Two reviewers (G.C.Y. and H.L.M.) independently assessed the risk of bias of the included studies based on the “Cochrane Handbook for Systematic Reviews of Interventions” imbedded in analysis software (9).

### Data synthesis and analysis

RevMan software (Version 5.2; Cochrane, Oxford, UK) was used for data synthesis. In order to avoid data entry errors, forest plots were performed through double-checked process by two reviewers (F.Y.L. and G.C.Y.). The change of each individual outcome was calculated by subtracting the pre-intervention value from the post-intervention value, and mean differences (MD) or standardized mean differences (SMD) with 95% confidence intervals (CIs) of studied outcomes between experimental and control groups were pooled using a random-effects model. Prior to data synthesis, data which was reported instead of mean and standard deviation (e.g., in case of median and range) were transformed to mean and standard deviation using the methods reported by Luo et al. (10) and Wan et al. (11). Statistical heterogeneity was estimated using the I2 statistic (12). The pooled outcomes are deemed having low statistical heterogeneity if *I*^2^ < 25%, moderate statistical heterogeneity if I2 ranged from 26 to 75%, and high statistical heterogeneity if *I*^2^ > 75%. The statistical significance was set at *p* < 0.05. Sensitivity analyses and funnel plots analysis for publication bias were not applicable due to the limited number of studies included in the meta-analysis.

## Results

### Search findings

**FIGURE 1:**
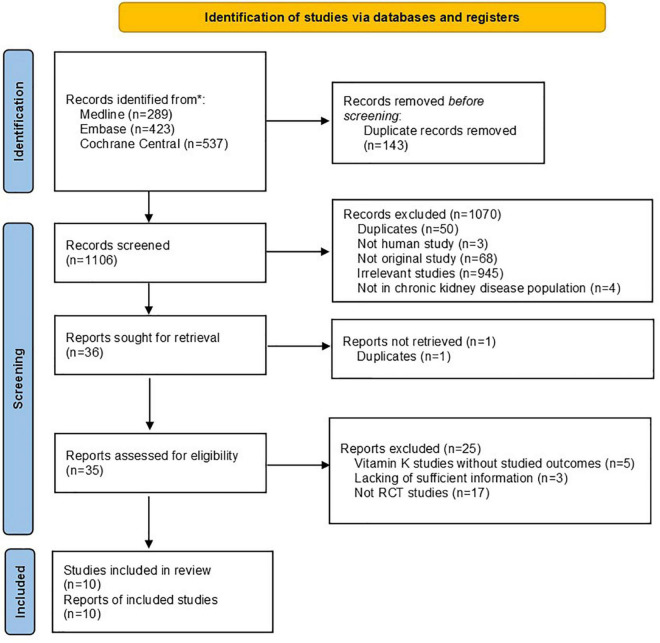
PRISMA flow chart of this systematic review.

A total of 1,106 citations were identified from literature searching after removing duplications. A total of 35 citations were kept for full text review after abstract screening and full text retrieval, among which 25 citations were further excluded. Therefore, ten RCT studies were finally included in the systematic review (see Figure 1).

### Study characteristics

In all, the ten RCTs involved 733 patients, including 365 in interventional group and 367 in control groups. Among these studies, six were conducted in hemodialysis patients (13–19), two were conducted in non-dialysis CKD patients (20, 21), and one was conducted in kidney transplantation recipients (22). All studies were conducted in adult population, except for one study which had been conducted in pediatric population. The percentages of male patients in individual studies ranged from 45.1 up to 71.6%. Two studies supplemented Vitamin K1, while the other eight studies supplemented Vitamin K2. Dosages of Vitamin K varied substantially from 90 μg/day up to 45 mg/day. Treatment durations also greatly varied from 3 months to 2 years. Characteristics of all included studies were shown in Table 1.

**TABLE 1:** Characteristics of included studies on the effect of vitamin K supplementation in chronic kidney disease.

References	Country	Registration	Participants	Number		Interventions			Vitamin K		VC related outcomes
				Total	Male, *n* (%)	Experimental	Control	Duration	Type	Dose	
De Vriese et al. (13)	Belgium	NCT02610933	Hemodialysis patients with AF treated with anticoagulation	88	63 (71.6%)	Vitamin K2 + rivaroxaban	Rivaroxaban	18 m	MK-7	2 mg Tiw	CA Agatston score, valve Agatston score, CA calcification volume score, valve calcification volume score, death
Kurnatowska et al. (20)	Poland	NCT01101698	Adult patients with CKD stages 3–5	42	22 (52.4%)	Vitamin K2 + cholecalciferol	Cholecalciferol	270 d	Vitamin K2	90 μg Qd	OC, CA Agatston score
Holden et al. (14)	Canada	NCT01528800	Hemodialysis patients	86	48 (55.2%)	Vitamin K1	Placebo	12 m	Phylloquinone	10 mg Tiw	dp-ucMGP, CA Agatston score, CA calcification volume score, AE, death
Lees et al. (22)	UK	ISRCTN22012044	Adults recipients with functioning kidney transplant for ≥ 1 year	90	63 (70.0%)	Vitamin K1	Placebo	12 m	Menadiol diphosphate	5 mg Tiw	dp-ucMGP, CA Agatston score, PWV, AE, death
Levy-Schousboe et al. (15)	Denmark	NCT02976246	Adult patient on hemodialysis ≥ 3 months	48	37 (77.1%)	Vitamin K2	Placebo	2 y	MK-7	360 μg Qd	dp-ucMGP, CA Agatston score, valve Agatston score, CA calcification volume score, valve calcification volume score, PWV, AE, death
Ochiai et al. (16)	Japan	N/A	Adult patient on hemodialysis > 2 years	40	18 (54.5%)	Vitamin K2	Null	12 m	Menatetrenone	45 mg Qd	OC, death
Oikonomaki et al. (17)	Greece	N/A	Adult hemodialysis patients	102	46 (45.1%)	Vitamin K2	Null	12 m	MK-7	200 μg Qd	dp-ucMGP, Aortic Agatston score, AE, death
Witham et al. (21)	UK	ISRCTN21444964	Adult patients at CKD stages 3–4	159	97 (61.0%)	Vitamin K2	Placebo	12 m	MK-7	400 μg Qd	OC, PWV, AE, death
Sarhan et al. (18)	Egypt	N/A	Adult patients on hemodialysis > 3 months	48	27 (56.3%)	Vitamin K2	Placebo	3 m	MK-7	90 μg Qd	dp-ucMGP, PWV
El Borolossy and El-Farsy (19)	Egypt	NCT04145492	Pediatric hemodialysis patients	30	18 (60.0%)	Vitamin K2	Null	4 m	MK-7	100 μg Qd	OC, dp-ucMGP

AE, adverse event; AF, atrial fibrillation; CA, coronary artery; CKD, chronic kidney disease; d, day; PWV, pulse wave velocity; m, months; n, number; N/A, not available; OC, osteocalcin; PWV, pulse wave velocity; Qd, once per day; Tiw, three times per week; VC, vascular calcification; y, year.

### Biochemical outcomes of vascular calcification

**FIGURE 2:**
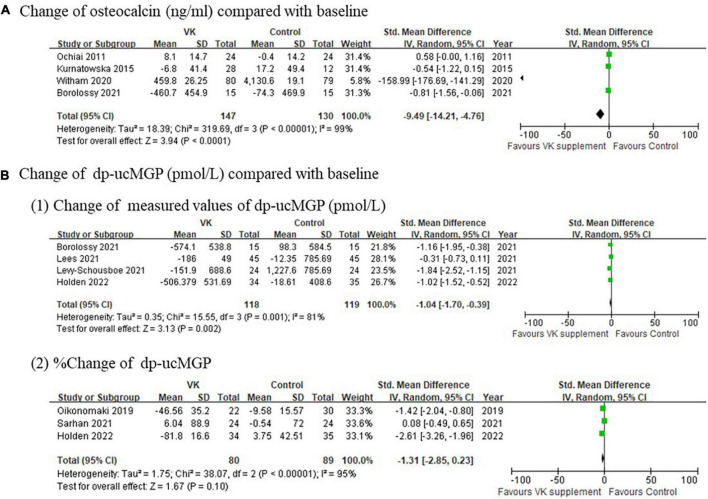
Pooled changes of **(A)** osteocalcin and **(B)** dp-ucMGP after Vitamin K supplementation compared with baseline.

The pooled results on biochemical measures indicated serum proteins relevant to vascular calcification decreased after vitamin K supplementation, including both OC (standardized mean difference of absolute changes: −9.49 ng/mL, 95% CI: −14.21 to −4.76 ng/mL, *I*^2^ = 99%, *p* < 0.0001) and dp-ucMGP (standardized mean difference of absolute changes: −1.04 pmol/L, 95% CI: −1.70 to −0.39 pmol/L, *I*^2^ = 81%, *p* < 0.0001; standardized mean difference of relative changes: −1.31%, 95% CI: −2.85 to 0.23%, *I*^2^ = 95%, *p* < 0.0001) (Figure 2), although extremely high heterogeneities existed for both indexes. These results supported the findings of decreased osteogenesis biomarkers after Vitamin K intervention observed in previously published studies.

#### Imaging outcomes of vascular calcification

**FIGURE 3:**
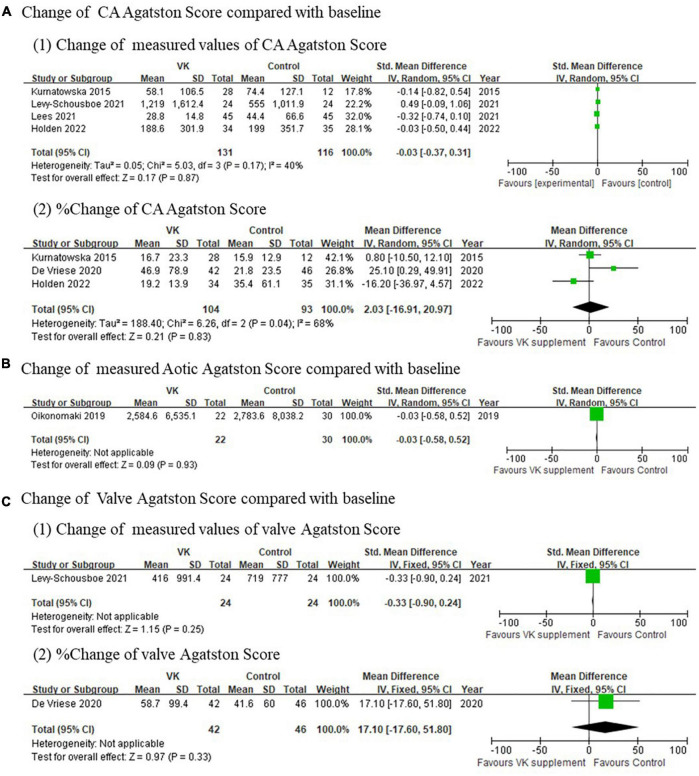
Pooled changes Agatston scores of coronary artery **(A)**, aortic artery **(B)**, and valve **(C)** after Vitamin K supplementation compared with baseline.

**FIGURE 4:**
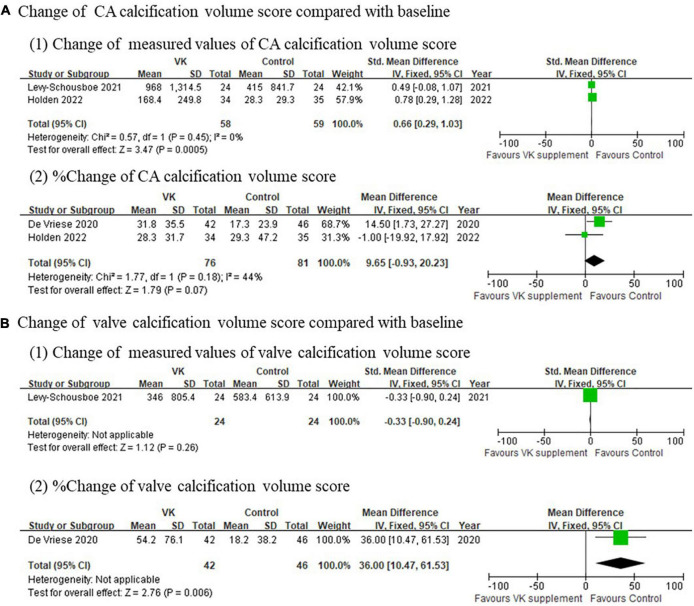
Pooled changes of calcification volume score of coronary artery and valve after Vitamin K supplementation compared with baseline. **(A)** Change of calcification volume score of coronary arteries and **(B)** change of calcification volume score of valves after Vitamin K supplementation compared with baseline.

Imaging outcomes of vascular calcification reported in individual studies exhibited inconsistent results. The changes of Agatston score for coronary artery (standardized mean difference of absolute changes: −0.03, 95% CI: −0.37 to 0.31, *I*^2^ = 40%, *p* = 0.17; mean difference of relative changes: 2.03%, 95% CI: −16.91 to 20.97%, *I*^2^ = 68%, *p* = 0.04), aortic artery (standardized mean difference of absolute changes: −0.03, 95% CI: −0.58 to 0.52), and valve (standardized mean difference of absolute changes: −0.33, 95% CI: −0.90 to 0.24; mean difference of relative changes:17.01%, 95% CI: −17.60 to 51.80%) showed no difference between Vitamin K supplementation and control groups (Figure 3), whereas the changes of calcification volume score of coronary artery (mean difference of absolute changes: 147.53, 95% CI: 63.77–231.29, *I*^2^ = 39%, *p* = 0.20; mean difference of relative changes: 9.65%, 95% CI: −0.93 to 20.23%, *I*^2^ = 44%, *p* = 0.18) and valve (standardized mean difference of absolute changes: −0.33, 95% CI: −0.90 to 0.241; mean difference of relative changes: 36.0%, 95% CI: 10.47–61.53%) partly favored the controls (Figure 4). However, this evidence was quite weak due to the limited number of studies included. Taken together, these findings failed to prove the effect of vitamin K to delay or prevent vascular calcification in CKD population from imaging perspective.

### Vascular elasticity

**FIGURE 5:**
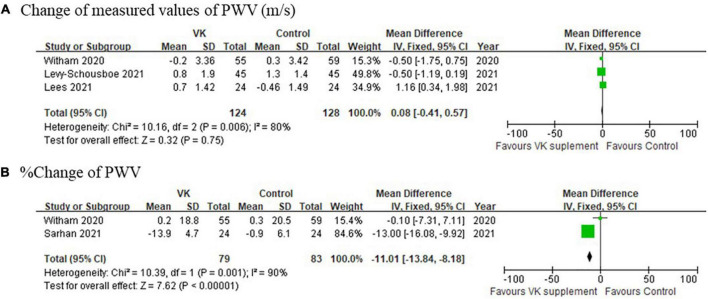
Pooled changes of pulse wave velocity after Vitamin K supplementation compared with baseline. **(A)** Absolute and **(B)** relative change of PWV after Vitamin K supplementation compared with baseline.

The pooled relative changes of PWV favored Vitamin K supplementation, whereas the pooled absolute changes did not support the differences (mean difference of absolute changes: 0.08 m/s, 95% CI: −0.41 to 0.57 m/s, *I*^2^ = 80%, *p* = 0.006; mean difference of relative changes: −11.01%, 95% CI: −13.84 to −8.18%, *I*^2^ = 90%, *p* = 0.001) (Figure 5). It should be noted that this finding was not solid due to high heterogeneities and limited number of studies included.

### Adverse events

**FIGURE 6:**
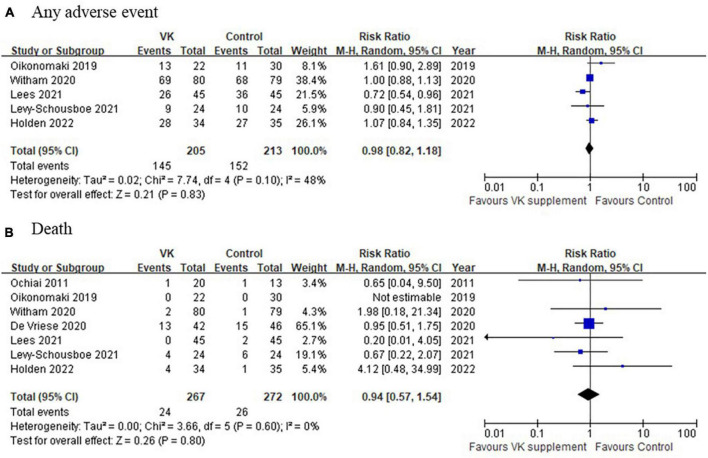
A comparison of adverse events between Vitamin K supplementation and control groups. **(A)** Any adverse event and **(B)** death between Vitamin K supplementation and control groups.

Pooled analysis of adverse events among the included studies indicated there was no significant difference in the incidence of adverse event between Vitamin K intervention and control groups (any adverse events: RR = 0.98, 95% CI: 0.82–1.18, *p* = 0.10, *I*^2^ = 48%, death: RR = 0.94, 95% CI: 0.57–1.54, *p* = 0.60, *I*^2^ = 0%) (Figure 6). These results consistently supported the safety of vitamin K supplementation in CKD population.

### Critical appraisal

**FIGURE 7:**
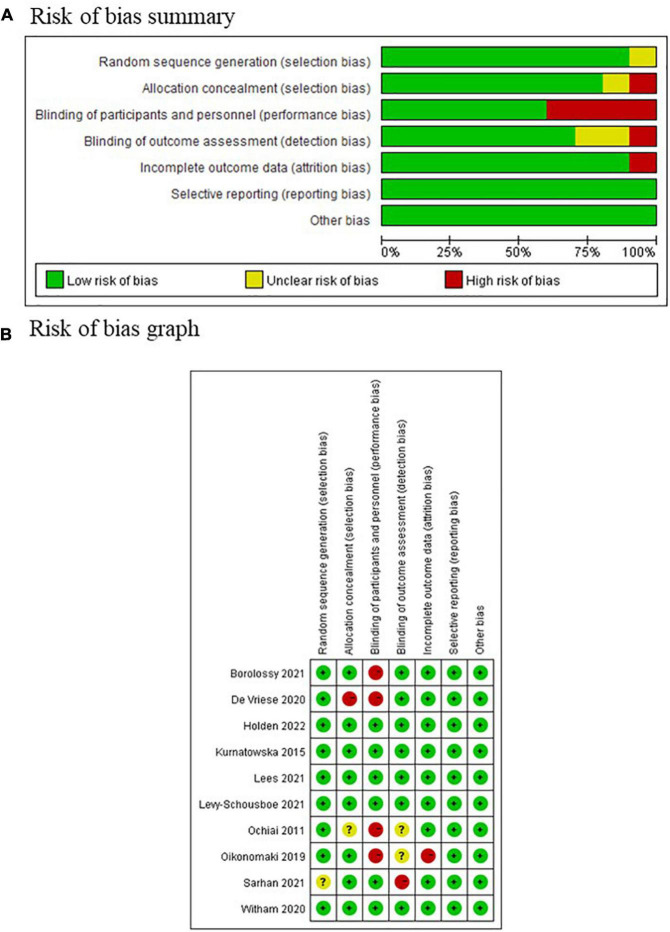
Risk of bias assessment of included study according to the Cochrane systematic review guidelines. **(A)** Risk of bias summary graph; **(B)** traffic light graph of risk of bias.

Five of the ten included studies were assessed as low risk of bias according to Cochrane systematic review guidelines, whereas the other five were assessed as high risk of bias (Figure 7). The domain with highest proportion of high risk was performance bias (4/10, 40%), i.e., blinding of participants and personnel, since these four studies utilized an open-label design.

## Discussion

The findings of this systematic review and meta-analysis indicated vitamin K supplementation helped to decrease serum biomarkers relevant to vascular calcification to a certain extent, might improve vascular elasticity reflected by PWV, however, failed to decrease calcification scores derived from radiology examinations. Half of the included studies had low risk of bias.

Despite of in-depth studies on potential mechanisms through which vitamin K supplementation protects against vascular calcification (23) and current and previous findings on the decrease of serum biomarkers relevant to vascular calcification after vitamin K intervention (24), there is insufficient data to support improvement of vascular calcification evidenced by radiology imaging, such as Agatston score and calcification volume score in our systematic review. Several possible reasons might explain this phenomenon. First, the current durations of vitamin K supplementation might be too short to observe obvious morphological changes, since vascular calcification is a long-term process of continuous progress. Second, there are many factors involved in the development and progression of vascular calcification in CKD, to name a few, serum phosphorus, serum calcium, serum parathyroid hormone, and dialysate calcium, and these factors closely relate to each other *via* complex crossover interactions. It is hardly possible to control or balance these factors throughout the study process. Therefore, theoretical improvement radiological outcomes, if existing, might be masked by effects from other confounders. Third, vitamin K supplementation might not be able reverse existing calcification lesions.

To the best of our acknowledgment, this is the first systematic review and meta-analysis that specifically addressed the value of vitamin K supplementation in CKD population and summarized the effects of vitamin K from biochemistry, imaging, and adverse effects perspectives. There are a number of ongoing trials on the effect of Vitamin K supplementation to delay or even stop vascular calcification in hemodialysis population (25–30) and pre-dialysis CKD population (31) (Table 2). Future results of these studies will help us better understand the value of vitamin K supplementation in the management of vascular calcification in CKD patients. It should be noted that vitamin K might have other clinical benefit other than preventing vascular calcification. It was reported vitamin K supplementation could reduce occurrence of muscle cramp in HD patients (32), possibly by improving vascular elasticity, as reflected in this systematic review. In addition, different doses of vitamin K supplementation were all very well tolerated in CKD patients. The net clinical benefits await future cost-effective research.

**TABLE 2:** A summary of ongoing clinical trials on the effect of Vitamin K supplementation to delay or even stop vascular calcification in CKD population.

References	Registration	Study design	Population	Sample size	Vitamin K		Control	Intervention	Duration	VC related outcomes
					Type	Dose				
Caluwe et al. (25)	NCT02610933	Randomized, open-label study	HD	117	MK-7	2,000 μg Tiw	Rivaroxaban		18 m	Calcification score of coronary arteries, thoracic aortic and valve; PWV
Haroon et al. (27)	NCT02870829	Randomized, open-label study	HD	200	MK-7	360 μg Tiw	Null		18 m	Coronary artery calcium score
Krueger et al. (29)	EudraCT No.: 2010-021264-14	Prospective, randomized, parallel, open-label, multicenter trial	HD	348	Phylloquinone	5 mg Tiw	Null	VK1	18 m	Calcification volume score of thoracic aortic and coronary artery
Oyama et al. (30)	UMINID000011490	Prospective, randomized, parallel group, multicenter trial	HD	200	Menatetrenone	45 mg Qd	Null	VK2	24 m	Abdominal aortic Agatston score
NCT (33)	NCT04900610	Multi-center, placebo-controlled, randomized, open-label trial	PD	120	MK-7	1 mg Qd	Placebo	VK2	1.5 y	PWV
NCT (34)	NCT04539418	Prospective, randomized, double-blind study	HD	59	Vitamin K2	2,000 μg Tiw IV	Saline	VK2	6 m	Intimate thickness of carotid artery
NCT (35)	NCT04477811	Randomized, triple-blind parallel study	HD	40	Menatetrenone, phytonadione	VK1 10 mg Tiw; VK2 90 μg Qd	Placebo	VK1; VK2	3 m	Serum ucMGP
NCT (36)	NCT03311321	Randomized, quadruple-blind parallel study	HD	60	MK-7	360 μg Qd	Placebo	VK2	8 w	FMD, PWV
NCT (37)	NCT02976246	Randomized, quadruple-blind parallel study	HD or PD	123	MK-7	360 μg Qd	Placebo	VK2	2 y	Coronary vascular and -valve calcification
NCT (38)	NCT02278692	Randomized, quadruple-blind parallel study	CUA	26	Phytonadione	10 mg Tiw	Placebo	VK1	12 w	Circulating MGP, area of CUA
NCT (39)	NCT01742273	Randomized, open-label study	HD	63	Phylloquinone	5 mg Tiw	Null	VK1	18 m	Calcification score of coronary arteries, thoracic aortic and valve

CUA, calcific uremic arteriolopathy; FMD, Flow-Mediated Dilation; HD, hemodialysis; m, month; PD, peritoneal dialysis; PWV, Pulse Wave Velocity; VC, vascular calcification; VK1, vitamin K1; VK2, vitamin K2; w, week; y, year.

There are a few limitations to be mentioned. First, the number of the included studies was too limited to allow sensitivity analysis or publication bias analysis. Second, the types of outcomes reported in the included bared great differences, weakening the power of horizontal comparisons. Future studies with standardized and consistent outcomes are needed to further clarify the value of vitamin K supplementation in this field.

## Conclusion

Based on the findings of this systematic review and meta-analysis, there is not yet solid evidence from RCTs to support the protective effects against vascular calcification of vitamin K supplementation in CKD population, however, improvement in biochemical markers of vascular calcification and slight decrease of vascular elasticity were observed. Future results of ongoing RCTs are needed to further elucidate the value of vitamin K supplementation against vascular calcification in CKD.

## Data availability statement

The original contributions presented in this study are included in the article/Supplementary material, further inquiries can be directed to the corresponding author.

## Author contributions

YF: conceptualization, project administration, and funding acquisition. YF and CG: methodology. CG and LH: software. CG, LH, and YF: data curation and formal analysis. YF, CG, LH, and LP: investigation. YF and LH: writing—original draft preparation. YF and LP: writing—review and editing. All authors have read and agreed to the published version of the manuscript, contributed to the article, and approved the submitted version.
