# Supplementary material for: Effects of vitamin K supplementation on vascular calcification in chronic kidney disease: A systematic review and meta-analysis of randomized controlled trials
Source: Front Nutr. 2023 Jan 10;9:1001826. doi: 10.3389/fnut.2022.1001826 (PMC9872197; doi:10.3389/fnut.2022.1001826)
Supplement: Supplementary file 1 [file Table_1.docx]

TableS1 Literature search strategies.

| Database | Strategies |
| --- | --- |
| Medline | ((vitamin K) OR phylloquinone OR menaquinones) AND ((chronic kidney disease) OR hemodialysis OR (end stage renal disease) OR (peritoneal dialysis) OR (renal disease*) OR (kidney disease*)) AND ((clinical trial) OR trial) |
| Embase | 1. vitamin K.mp.  2. phylloquinone.mp.  3. menaquinones.mp.  4. 1 OR 2 OR 3  5. chronic kidney disease/  6. hemodialysis/  7. end stage renal disease/  8. peritoneal dialysis/  9. renal disease*.mp.  10. kidney disease*.mp.  11. 5 OR 6 OR 7 OR 8 OR 9 OR 10  12. trial.mp.  13. clinical trial.mp.  14. 12 OR 13  15. 4 AND 11 AND 14 |
| Cochrane Central Library | ((vitamin K) OR phylloquinone OR menaquinones) AND ((chronic kidney disease) OR hemodialysis OR heamodilaysis OR (end stage renal disease) OR (peritoneal dialysis) OR (renal disease*) OR (kidney disease*)) AND ((clinical trial) OR trial) |
